# Treatment of Ipilimumab Induced Graves' Disease in a Patient with Metastatic Melanoma

**DOI:** 10.1155/2016/2087525

**Published:** 2016-01-11

**Authors:** Umal Azmat, David Liebner, Amy Joehlin-Price, Amit Agrawal, Fadi Nabhan

**Affiliations:** ^1^Division of Endocrinology, Diabetes, and Metabolism, The Ohio State University, Columbus, OH, USA; ^2^Division of Medical Oncology and Department of Biomedical Informatics, The Ohio State University, Columbus, OH, USA; ^3^Department of Pathology, The Ohio State University, Columbus, OH, USA; ^4^Department of Otolaryngology, Head and Neck Surgery, The Ohio State University, Columbus, OH, USA

## Abstract

*Objective*. Thyroid disease has been reported among the endocrinopathies that can occur after treatment with ipilimumab. Graves' disease, however, has been rarely reported with this medication. Here we report a case of Graves' disease diagnosed after initiation of ipilimumab in a patient with melanoma.* Methods*. We present the clinical presentation and management course of this patient followed by a related literature review.* Results*. A 67-year-old male with metastatic melanoma was started on ipilimumab. He developed hyperthyroidism after two doses of ipilimumab. The cause of hyperthyroidism was determined to be Graves' disease. Ipilimumab was held and the patient was started on methimazole with return to euthyroid status. Ipilimumab was resumed and the patient continued methimazole during the course of ipilimumab therapy, with controlled hyperthyroidism. Restaging studies following four cycles of ipilimumab showed complete response in the lungs, with residual melanoma in the neck. The patient then underwent total thyroidectomy and left neck dissection as a definitive treatment for both hyperthyroidism and residual melanoma.* Conclusion*. Graves' disease can develop after starting ipilimumab and methimazole can be an effective treatment. For patients whose hyperthyroidism is well-controlled on methimazole, ipilimumab may be resumed with close monitoring.

## 1. Introduction

Ipilimumab is an FDA-approved human monoclonal antibody that blocks an immune checkpoint molecule called cytotoxic T-lymphocyte-associated antigen 4 (CTLA-4) leading to increased antitumor activity of tumor-specific T-cells and improved survival in patients with melanoma [1]. However, it is associated with risk of autoimmune toxicities, including endocrinopathies such as hypophysitis, hypothyroidism, and thyroiditis [2, 3]. Graves' disease also has been rarely reported with this medication [4–7]. We present a case in which Graves' disease was diagnosed after starting a patient with metastatic melanoma on ipilimumab and discuss the clinical presentation and therapeutic interventions.

## 2. Case Report

**Figure 1:**
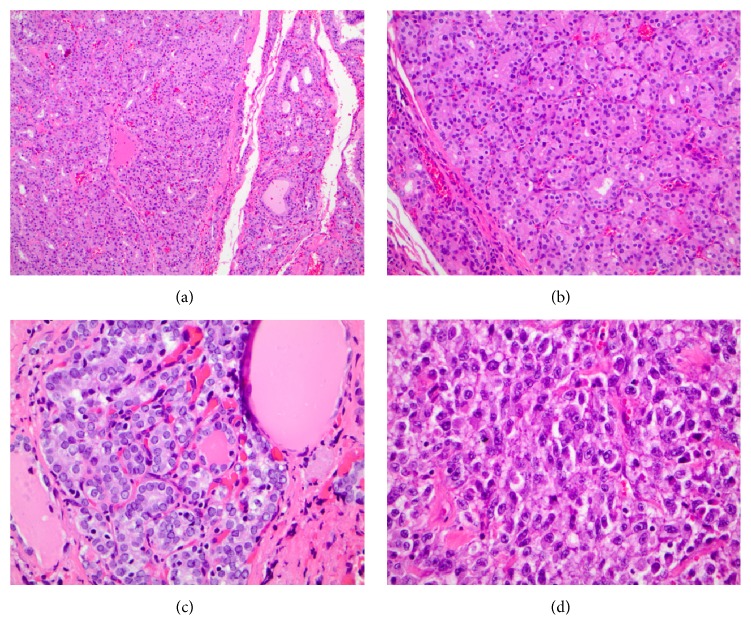
Nodular hyperplasia of the thyroid (a) secondary to the patient's Graves' disease, demonstrating abundant follicular structures with scant colloid (b); high power view of patient's papillary thyroid microcarcinoma demonstrating vesicular nuclei, nuclear grooves, and nuclear crowding (c); and representative discohesive, high grade malignant cells of the patient's malignant melanoma requiring ipilimumab therapy (d).

The patient is a 67-year-old male with metastatic melanoma involving cervical lymph nodes and lungs. He had normal thyroid function tests before initiation of ipilimumab and he has no previous history of thyroid disease. Ipilimumab was started at a dose of 3 mg/kg every three weeks. After receiving two of four planned cycles of therapy, he developed clinical and biochemical hyperthyroidism (Table 1). There was no thyroid tenderness on exam and no palpable thyroid nodules. There were also no signs of ophthalmopathy. Laboratories revealed an elevated thyroid stimulation immunoglobulin level and I-123 scan revealed diffuse homogeneous uptake that was elevated at 6 hours at 30.4% (normal is 5–15%) and at 24 hours at 47.4% (normal 10–33%), consistent with Graves' disease. Ipilimumab was held, and the patient was started on methimazole at a dose of 30 mg/day with titration to control the thyroid hormone levels (Table 1). The highest dose of methimazole used was a total of 35 mg a day. Restaging CT scans showed persistent cervical adenopathy, but resolution of his lung nodules consistent with an immune response to ipilimumab. Given the excellent early clinical response to ipilimumab and the desire to achieve the greatest presurgical response, it was recommended that he complete all 4 cycles of ipilimumab if his hyperthyroidism could be safely controlled. He subsequently received two additional cycles of ipilimumab on methimazole to complete the treatment plan for the melanoma. Methimazole was continued during this time and hyperthyroidism remained controlled (Table 1). He subsequently underwent a left neck dissection for residual metastatic melanoma along with total thyroidectomy. Pathology (Figure 1) revealed nodular and papillary hyperplasia of the thyroid, common findings in Graves' disease, along with an incidental papillary thyroid microcarcinoma. The patient was started on levothyroxine after surgery and his thyroid function tests normalized (Table 1).

## 3. Discussion

Ipilimumab is an immune therapy that has been shown to increase survival in patients with melanoma [1]. Ipilimumab works by blocking CTLA-4, which is an immune checkpoint receptor expressed on the surface of helper T-cells. CTLA-4 normally functions to impair the costimulatory activation of T-cells by CD28, leading to downregulation of T-cell activity. By blocking CTLA-4, ipilimumab removes this negative regulation and induces immune responses that can lead to antitumor activity.

Ipilimumab has been associated with the development of new autoimmune endocrinopathies, likely related directly to its mechanism of action. The most common endocrine side effect is hypophysitis with an incidence rate of 11% in one study [8] and 8% in another [2]. Ipilimumab can lead to autoimmune thyroid disease, with the most common manifestation being hypothyroidism in about 6% followed by thyroiditis characterized by hyperthyroid and hypothyroid phases [2]. Hyperthyroidism resulting from overproduction of thyroid hormone as seen in Graves's disease has been more rarely reported. One case of thyroid storm was reported by Yu et al. [9] in a patient receiving ipilimumab, which occurred after two doses of ipilimumab and subsequently responded to antithyroid medication. Other studies reported eye disease typical of Graves's disease after using ipilimumab [4–7]. In one of these cases [6], hyperthyroidism developed in addition to the eye disease. The diagnosis of Graves' disease was confirmed in our patient given his elevated thyroid stimulating immunoglobulin, which has very high specificity for diagnosis of Graves' disease [10]. This was also supported by elevated iodine uptake and a homogenous scan, which further confirmed Graves's disease [11]. Pathologically, the nodular hyperplasia noted at the time of resection was supportive of the diagnosis as well.

It is difficult to characterize a typical time-course for the development of ipilimumab-related Graves' disease due to the small number of cases. McElnea et al. [4] reported the case of ophthalmopathy in a patient who was euthyroid after two doses while Min et al. [5] reported Graves' eye disease after four doses of ipilimumab. These are similar to the present case in which the disease developed after two doses of ipilimumab.

The pathogenesis of Graves' disease is due to the development of activating antibodies directed against the TSH receptor, which results in hyperthyroidism. This antibody formation involves activation of T2 helper cells. Therefore it is plausible to hypothesize that blocking CTLA-4 results in the development of activating antibodies against the TSH receptor thereby causing Graves' disease in susceptible individuals.

There are several points that support this relation between CTLA-4 and Graves' disease. Heward et al. [12] showed allelic association between the G allele of the CTLA-4 gene and Graves' disease. Furthermore in the same study [12] there was a relationship between allelic variation of the CTLA-4 gene and circulating free T4 concentration at the time of diagnosis suggesting a link between the gene and the severity of Graves' disease. Daroszewski et al. [13] showed that serum levels of a transcript of CTLA-4 were increased in patients with Graves' disease.

Management of patients with Graves's disease in patients being treated with ipilimumab is not well described. It is recommended that ipilimumab be stopped for any endocrinopathy that is not controlled [14]. In our case, ipilimumab was initially held and hyperthyroidism was controlled with methimazole. Then ipilimumab was restarted with successful control of thyroid hormone levels throughout the remainder of the treatment course with methimazole. The choice to perform surgery in this patient was influenced by the fact that radical neck surgery was already indicated for treatment of his melanoma. Other treatment options such as radioactive iodine treatment or continuation of methimazole were also valid choices for our patient. It is also possible that Graves' disease would remit over time after completion of the ipilimumab therapy. Following TSH receptor antibodies to undetectable levels after taking methimazole can help in predicting Graves' disease remission [11].

It has not yet been determined whether it is possible to predict the development of Graves' disease after starting ipilimumab. Measuring thyroid stimulating immunoglobulins may be helpful; however these may not be elevated unless the patient develops hyperthyroidism. Given the challenge in predicting Graves' disease, it is important to monitor all patients receiving this medication by measuring thyroid function regularly.

In summary Graves's disease can develop after starting ipilimumab. Managing these patients can be challenging, especially if further doses of ipilimumab are recommended, and this management has not been well described in the literature. As in our case, methimazole appears to be efficacious in this treatment, as it would be in the more commonplace cases of Graves's disease developing without the use of ipilimumab.

## Figures and Tables

**Table 1:** Thyroid function tests changes during treatments.

	Before ipilimumab	1 month after ipilimumab	Ipilimumab held, methimazole started	2 months after ipilimumab	Ipilimumab restarted	1 month after restarting ipilimumab	1 month after total thyroidectomy
TSH (0.55–4.78 mUJ/mL)	1.561	0.009	<0.008	0.015	0.015	0.071	0.892
Free T4 (0.89–1.76 ng/dL)	1.42	3.38	3.64	1.36	1.31	1.01	1.47
Free T3 (2/3–4.2 pg/mL)		8.8	9.8	4.1	4.1	3.6	
TSI (thyroid stimulating immunoglobulin) (<140%)		368					

## References

[1] Hodi F. S., O'Day S. J., McDermott D. F. (2010). Improved survival with ipilimumab in patients with metastatic melanoma. *The New England Journal of Medicine*.

[2] Ryder M., Callahan M., Postow M. A., Wolchok J., Fagin J. A. (2014). Endocrine-related adverse events following ipilimumab in patients with advanced melanoma: a comprehensive retrospective review from a single institution. *Endocrine-Related Cancer*.

[3] Corsello S. M., Barnabei A., Marchetti P., De Vecchis L., Salvatori R., Torino F. (2013). Endocrine side effects induced by immune checkpoint inhibitors. *The Journal of Clinical Endocrinology & Metabolism*.

[4] McElnea E., Ní Mhéalóid Á., Moran S., Kelly R., Fulcher T. (2014). Thyroid-like ophthalmopathy in a euthyroid patient receiving ipilimumab. *Orbit*.

[5] Min L., Vaidya A., Becker C. (2011). Thyroid autoimmunity and ophthalmopathy related to melanoma biological therapy. *European Journal of Endocrinology*.

[6] Borodic G. E., Hinkle D. (2014). Ipilimumab-induced orbital inflammation resembling graves disease with subsequent development of systemic hyperthyroidism from CTLA-4 receptor suppression. *Ophthalmic Plastic & Reconstructive Surgery*.

[7] Sohrab M. A., Desai R. U., Chambers C. B., Lissner G. S. (2013). Re:‘drug-induced graves disease from CTLA-4 receptor suppression’. *Ophthalmic Plastic & Reconstructive Surgery*.

[8] Faje A. T., Sullivan R., Lawrence D. (2014). Ipilimumab-induced hypophysitis: a detailed longitudinal analysis in a large cohort of patients with metastatic melanoma. *The Journal of Clinical Endocrinology & Metabolism*.

[9] Yu C., Chopra I. J., Ha E. (2015). A novel melanoma therapy stirs up a storm: ipilimumab-induced thyrotoxicosis. *Endocrinology, Diabetes & Metabolism Case Reports*.

[10] Barbesino G., Tomer Y. (2013). Clinical utility of TSH receptor antibodies. *The Journal of Clinical Endocrinology & Metabolism*.

[11] Bahn R. S., Burch H. B., Cooper D. S. (2011). Hyperthyroidism and other causes of thyrotoxicosis: management guidelines of the American Thyroid Association and American Association of Clinical Endocrinologists. *Thyroid*.

[12] Heward J. M., Allahabadia A., Armitage M. (1999). The development of Graves' disease and the CTLA-4 gene on chromosome 2q33. *The Journal of Clinical Endocrinology and Metabolism*.

[13] Daroszewski J., Pawlak E., Karabon L. (2009). Soluble CTLA-4 receptor an immunological marker of Graves' disease and severity of ophthalmopathy is associated with CTLA-4 Jo31 and CT60 gene polymorphisms. *European Journal of Endocrinology*.

[14] Fecher L. A., Agarwala S. S., Stephen Hodi F., Weber J. S. (2013). Ipilimumab and its toxicities: a multidisciplinary approach. *The Oncologist*.

